# Management of Severe Hypertriglyceridemia in Pregnancy With Niacin: Reevaluating Safety and Therapeutic Benefits

**DOI:** 10.1155/crie/2644678

**Published:** 2025-01-30

**Authors:** Nisha Suda, Daisy Leon-Martinez, Patricia R. Peter, Clare A. Flannery, Roxanna A. Irani

**Affiliations:** ^1^Department of Medicine, Section of Endocrinology and Metabolism, Albert Einstein College of Medicine–Montefiore Medical Center, Bronx, New York, USA; ^2^Department of Obstetrics, Gynecology and Reproductive Sciences, University of California at San Francisco, San Francisco, California, USA; ^3^Department of Internal Medicine, Section of Endocrinology and Metabolism, Yale School of Medicine, New Haven, Connecticut, USA; ^4^Department of Obstetrics, Gynecology and Reproductive Sciences, Yale School of Medicine, New Haven, Connecticut, USA

**Keywords:** case report, hypertriglyceridemia, niacin, pancreatitis, pregnancy

## Abstract

**Background:** Severe hypertriglyceridemia (triglycerides (TGs) >1000 mg/dL, >11.3 mmol/L) is a rare but potentially morbid condition in pregnancy. Physiological changes in pregnancy may unmask or exacerbate an underlying defect in TG metabolism. When conventional therapies are ineffective in controlling TG levels, a personalized management approach is needed. We present a case of severe hypertriglyceridemic pancreatitis successfully managed with niacin, a treatment that has seen limited use in pregnancy due to the paucity of available data.

**Case Presentation:** A 29-year-old pregnant woman with a history of cholecystectomy and a prepregnancy BMI of 30.6 kg/m^2^ presented at 12 weeks' gestation with acute pancreatitis and severe hypertriglyceridemia (6900 mg/dL, 77.9 mmol/L). After initial management with intravenous (IV) fluids, insulin infusion, and a low-fat diet, her TG levels improved. However, she was readmitted at 23 weeks' gestation with recurrent hypertriglyceridemia (2872 mg/dL, 32.4 mmol/L), requiring a more aggressive insulin regimen. Despite various interventions, including omega-3 fatty acids (O3FAs), fenofibrate, and central venous catheter insulin infusion, her TG levels remained elevated, necessitating early delivery at 34 weeks' gestation. Her postpartum recovery included continued TG management with fenofibrate and O3FAs. Four years later, during a second pregnancy, she presented with similar hypertriglyceridemia, managed with diet, metformin, fenofibrate, and insulin. Due to persistent hypertriglyceridemia (>3000 mg/dL, 33.9 mmol/L), niacin was added as an additional therapy and titrated to 2000 mg/day, which successfully sustained TG levels below 1000 mg/dL (11.3 mmol/L) through the remainder of her pregnancy. She delivered her second child via cesarean section at 35 weeks' gestation due to preeclampsia. Both children had developmental issues, with her first child diagnosed with attention-deficient hyperactivity disorder (ADHD) and her second child with autism spectrum disorder and motor delays. The patient was encouraged to remain on long-term management for her metabolic condition.

**Conclusions:** Managing severe hypertriglyceridemia during pregnancy is challenging due to uncertainties about treatment efficacy and safety. Timely reduction of maternal TGs is essential to prevent complications and requires adjustments throughout pregnancy. This case demonstrates the effectiveness and safety of niacin, often underutilized due to perceived side effects, in managing severe hypertriglyceridemia in pregnancy when other treatments were inadequate.

## 1. Introduction

Severe hypertriglyceridemia (triglycerides (TGs) >1000 mg/dL, >11.3 mmol/L) in pregnancy is rare and remains challenging to manage. Significant risks to mother and fetus include hyperviscosity syndrome, pancreatitis, preeclampsia, preterm labor, prematurity, and death [1–5].

**Figure 1:**
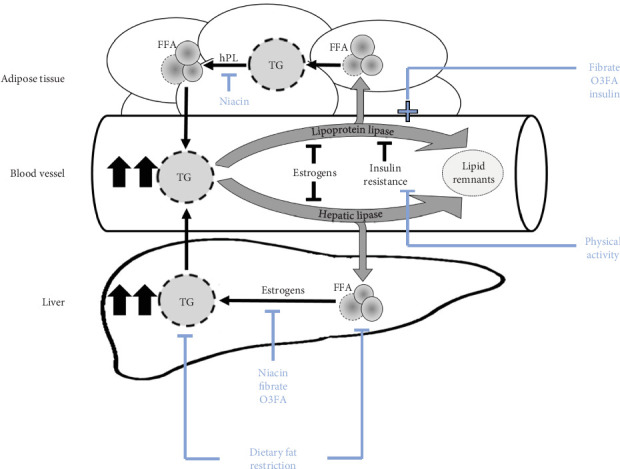
Impact of pregnancy hormones on maternal TG levels and therapy interventions. Schematic diagram illustrating the pathways upon which estrogens and hPL act to increase TG levels. Lipase enzymes hydrolyze circulating TG, allowing FFAs to be taken into the liver or other organs such as adipose tissue. Estrogens increase liver synthesis of TGs and decrease the clearance of circulating TGs by inhibiting the activity of hepatic lipase and LpL. This inhibition of lipase activity results in higher circulating TG levels. In the third trimester, hPL promotes lipolysis of maternal adipose tissue to further increase circulating TG levels. Furthermore, hPL also increases insulin resistance. This resistance prevents insulin from activating LpL, thus ensuring higher circulating TG levels. *FFA*, free fatty acids; hPL, human placental lactogen; LpL, lipoprotein lipase; TG, triglycerides.

TGs will increase two- to fourfold during normal pregnancy, peaking in the third trimester, though rarely exceeding approximately 300 mg/dL (~3.4 mmol/L) [6]. This adaptive increase provides rapidly accessible energy for the fetus during maternal fasting. While lipogenesis and increased fat storage predominate early pregnancy, rising levels of estrogens in the second trimester promote increases in circulating maternal cholesterol, phospholipids, and TGs. Estrogens increase liver synthesis of TGs [7, 8] and inhibit hepatic lipase and lipoprotein lipase (LpL) activity, thus, reducing TG clearance. In the third trimester, human placental lactogen (hPL) promotes lipolysis of stored TG in maternal adipose tissue and increases insulin resistance, limiting insulin stimulation of LpL, thus, maintaining elevated TG levels (Figure 1). These physiological changes in pregnancy may further exacerbate TG levels in someone with an underlying defect in TG metabolism [9].

Pregnant patients with preexisting disorders of lipoprotein metabolism may experience persistent TG levels greater than 1000 mg/dL (11.3 mmol/L). Genetic abnormalities are often linked to these disorders, though patients may have a mixed clinical picture [10, 11]. Lowering maternal TG levels helps reduce complications [2, 12, 13], but management remains challenging due to limited therapeutic data in pregnancy and the absence of established treatment guidelines. While recent years have seen an increase in published case reports, they have not led to formal guidelines, and few highlight the effectiveness of niacin in reducing TG levels. Presently, management of hypertriglyceridemia in pregnancy includes lifestyle changes, medications, and/or plasmapheresis (Table 1).

## 2. Case Presentation

**Figure 2:**
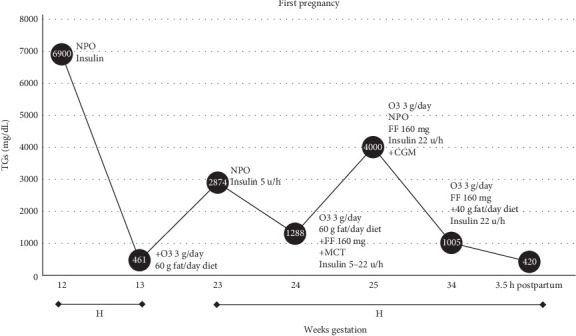
Triglyceride (TG) values throughout first pregnancy. Values for this patients' TG levels and weeks' gestation with noted onboard therapies, initiated therapies (added therapies noted with “+”), and associated events are detailed here. Inpatient hospital admission duration noted on *x*-axis bars with “H.” “Insulin” is insulin infusion with dextrose D10 concentration clamp targeted postprandial glucose 90–140 mg/dL (1.0–1.6 mmol/L), steady state insulin infusion rates indicated in units per hour (u/h). CGM, continuous subcutaneous glucose monitor; FF, fenofibrate; MCT, medium chain TGs; NPO, oral restriction excluding medications; O3, omega-3 fatty acid fish oil.

A 29-year-old woman at 12 weeks' gestation presented with a 2-day history of severe epigastric pain, nausea, and vomiting. Her medical history was notable for a cholecystectomy and prepregnancy BMI of 30.6 kg/m^2^ without known metabolic complications. She did not take any medications, nor use cigarettes, alcohol, or drugs. She was physically active in her work at a retail store, but did not follow a specific exercise or dietary regimen. Her family history included hyperlipidemia in her mother and maternal grandmother, but neither family member experienced lipid exacerbations in pregnancy or had undergone genetic testing for familial hypertriglyceridemia. On admission, her vitals were within normal limits. Exam was notable for a gravid uterus, tenderness to palpation in the epigastrium, and left upper quadrant, with no acanthosis, striae, jaundice, or xanthomas. Significant labs (Figure 2) included TGs of 6900 mg/dL (78.0 mmol/L; normal < 150 mg/dL, <1.7 mmol/L) and lipase of 67 U/L (normal <60 U/L). MRI confirmed acute pancreatitis. After 4 days of oral intake restriction, intravenous (IV) fluids, antiemetics, and continuous peripheral IV insulin infusion, her symptoms improved to a lipase 24 U/L and TGs 461 mg/dL (5.2 mmol/L). The insulin infusion was supplemented with IV dextrose at a concentration of 5 g dextrose per 100 mL fluid (D5) and provided by peripheral venous access, which was changed regularly per hospital protocol. She was discharged to home on a low-fat diet (60 g fat daily) and omega-3 fatty acids (O3FAs) 3000 mg daily, as they were therapeutic interventions with demonstrated safety and efficacy in pregnancy [11, 14].

At 23 5/7 weeks' gestation, she was readmitted with similar epigastric pain, with TGs 2872 mg/dL (32.4 mmol/L), normal lipase and liver function tests, and an abdominal ultrasound notable for hepatic steatosis. The patient's hypertriglyceridemia was managed again with a continuous insulin IV infusion via peripheral venous access, O3FA, and fat restricted diet. Due to persistently elevated TG levels, medium chain TG oils were added [14], but quickly discontinued due to nausea (Figure 1); therefore, fenofibrate 160 mg once daily was added as its use was demonstrated in the obstetrics and endocrine literature [11, 14, 15]. Despite these treatments, TGs continued to climb to 4601 mg/dL (52.0 mmol/L). A central venous catheter was placed when apheresis was considered, however, that intervention was deferred due to potential risks and poor durability of the therapy [16]. Unfortunately, pancreatitis reoccurred; therefore, oral intake was held and a continuous insulin infusion through the central venous catheter with a dextrose clamp was initiated. To target a postprandial glucose range of 90–140 mg/dL (1.0–1.6 mmol/L), 10 g dextrose per 100 mL fluid (D10) was administered at a median rate of 70–90 cc/h. Given the ongoing need for hourly blood sugar monitoring, a subcutaneous continuous glucose monitor was placed. By the third trimester, she required 500 units of IV insulin per day with a maximum D10 rate of 110 cc/h. Her TGs stabilized at a median level of 1288 mg/dL (14.6 mmol/L), without further episodes of pancreatitis. The patient was induced at 34 0/7 weeks' gestation due to persistent hypertriglyceridemia. Antenatal corticosteroids were administered along with escalating rates of IV insulin, up to 22 u/h with D10. After a failed induction of labor, she delivered a healthy baby boy by cesarean section at 34 3/7 weeks' gestation, weighing 2156 g with Apgar score 9/9 and normal metabolic parameters without hypothermia or hypoglycemia. Postpartum, the insulin infusion was stopped, and TG levels dropped from 1005 mg/dL (11.4 mmol/L) to 420 mg/dL (4.7 mmol/L) within 3.5 h. The neonate was appropriately grown for gestational age, had an uneventful hospital course, and was discharged to home 11 days after birth. The patient was discharged to home on postpartum day four on fenofibrate 160 mg daily and O3FA 3 g daily. By 6 weeks postpartum, her TG levels were 150 mg/dL (1.7 mmol/L).

Lipoprotein electrophoresis suggested a diagnosis of Frederickson Type IV familial hypertriglyceridemia, but genetic testing was negative for its common associated mutations. Genetics revealed heterozygous null variants in two genes (*ABCG8* and *PPARG*) that were relevant to her hypertriglyceridemia phenotype, but were of uncertain significance; therefore, no change in therapy was recommended. She continued to follow with endocrinology outpatient, where her TGs remained less than 250 mg/dL (2.8 mmol/L) on fenofibrate 160 mg daily and O3FA 3 g daily. Metformin was initiated for prediabetes, and initiating a nonhormonal or progestin-only contraception was discussed. Patient did not return for initiation of contraceptives.

**Figure 3:**
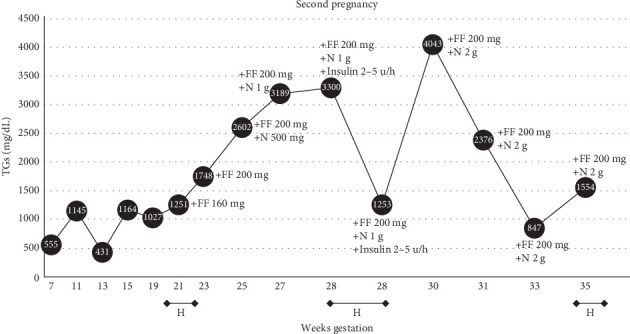
Triglyceride (TG) values throughout second pregnancy. Values for this patients' TG levels and weeks' gestation with noted onboard therapies, initiated therapies (added therapies noted with “+”), and associated events are detailed here. Throughout the second pregnancy, she was maintained on metformin 1500 mg daily, omega-3 fatty acids 2 g twice daily, and a 40 g/day low-fat diet. Inpatient hospital admission duration noted on *x*-axis bars with “H.” “Insulin” is insulin infusion with dextrose 10% clamp targeting postprandial glucose 90–140 mg/dL (1.0–1.6 mmol/L), steady state insulin infusion rates indicated in units per hour (u/h). FF, fenofibrate; N, niacin; O3, omega-3 fatty acid fish oil. Corresponding mmol/L units are noted in the text.

Four years later, after being lost to follow-up, she presented to the endocrine clinic at 7 weeks' gestation with her second pregnancy. She was taking O3FA 3 g and metformin 1500 mg with TG 555 mg/dL (6.3 mmol/L). A more stringent fat-restricted diet (<40 g/day) was emphasized. At 13 weeks' gestation, she was diagnosed with diabetes following an oral glucose tolerance test, which showed a blood sugar of 206 mg/dL (11.3 mmol/L) 1 h after a 50 g glucose load. She was started on basal insulin 20 units/day. Through the second trimester, she was asymptomatic but her TGs increased over >1000 mg/dL (>11.3 mmol/L). Fenofibrate was reinitiated (Figure 3). Despite fibrate titration, by 25 weeks' gestation, TGs were 2600 mg/dL (29.4 mmol/L). As the previous therapies failed to prevent recurrent pancreatitis in her first pregnancy, the addition of niacin 500 mg once per day was recommended after careful consideration with the patient [17, 18]. Two weeks later, niacin was increased to 1 g per day. Although she remained asymptomatic, she was readmitted for insulin infusion as a precaution when TGs continued to increase over 3000 mg/dL (33.9 mmol/L). She was discharged to home with a TG level of 1253 mg/dL (14.1 mmol/L) on fenofibrate 2 g and niacin 1 g daily. At 30 weeks' gestation TGs increased to >4000 mg/dL (45.2 mmol/L), and in the absence of symptoms, she declined hospital admission. Higher doses of niacin were offered and she started niacin 2000 mg/day which led to sustained improvement in TG levels to 847 mg/dL (9.6 mmol/L) through the remainder of her pregnancy. Diabetes was well controlled on metformin 1500 mg per day and basal insulin 26 units/day. At 35 6/7 weeks' gestation her home blood pressure was 170/100 mmHg, and she was seen in the outpatient clinic where she was evaluated for a headache and scotomata. She was admitted to the hospital and treated with IV labetalol for high blood pressure, and subsequently underwent a repeat cesarean delivery at 35 6/7 weeks' gestation due to preeclampsia with severe features. She delivered a 3600 g baby boy with Apgar score 7/9, and without birth defects. After a 6-day neonatal ICU stay for hypothermia and hypoglycemia, the baby was safely discharged to home with evidence of normal growth trajectory. The patient was discharged to home on niacin 2000 mg daily, fibrate 200 mg daily, and O3FA 2 g twice per day, with plans to adjust medications in the outpatient setting and discuss nonestrogen contraceptive methods; however, she was lost to follow-up for 1.5 years. Off all medications, TG levels were 150 mg/dL (1.7 mmol/L), with an Hgb A1c 7.3%, and she had not followed-up with gynecology for initiation of contraception.

Six years after her initial presentation, the patient reported that her first child had normal development but was diagnosed with attention-deficient hyperactivity disorder (ADHD) for which he is following with a therapist. Her second child has motor and speech delays which neurology attributed to a “complex neurodevelopmental disorder of unclear etiology” and autism spectrum disorder, with a genetic variant of uncertain significance in the scaffolding gene (*MPD2*).

## 3. Discussion

We report niacin's role in achieving safe, effective, and sustained TG control during pregnancy. While limited data for niacin's use in pregnancy has hampered its use [1, 11]. Our patient experienced a 79% decrease in TG levels to less than 1000 mg/dL (11.3 mmol/L) on niacin 2 g/day, thus, greatly reducing the risk of pancreatitis and hospitalization. This robust response suggests that her specific metabolic defect was responsive to niacin.

Pancreatitis is known to occur during pregnancy with 12% of cases occurring during the first and second trimester, 50% in the third trimester, and is more common in multiparous women [18, 19]. Of the various causes of pregnancy-associated pancreatitis, 5% are estimated to be from hypertriglyceridemia and can be life-threatening to the patient and child [1, 18]. In addition to hypertriglyceridemic pancreatitis, our patient had preeclampsia which is also associated with hypertriglyceridemia. Hypertriglyceridemia increases inflammation, hypercoagulability, and endothelial dysfunction, putting patients at higher risk of placental vasculopathy, and thus, could contribute to the similar pathophysiological changes resulting in pre-eclampsia [20].

TGs have a normal two- to fourfold increase in pregnancy, peaking in the third trimester, rarely exceeding 250–332 mg/dL (2.82–3.75 mmol/L) [18]. This patient's atypical presentation of hypertriglyceridemia and pancreatitis in the first trimester of her initial pregnancy should have prompted consideration of an underlying and more serious condition. Later investigation into the cause of the severe hypertriglyceridemia was conducted through lipoprotein electrophoresis, which revealed elevated pre-beta lipoproteins, indicative of Frederickson Type IV familial hypertriglyceridemia. This disorder is characterized by increased very low density lipoprotein (VLDL) production, chylomicrons, and moderate TG levels (200–500 mg/dL, 2.3–5.6 mmol/L), posing a significant risk for pancreatitis. It has been reported that pregnant women with this condition can experience hypertriglyceridemic pancreatitis [21]. Subsequent genetic testing did not reveal an LpL deficiency; however, it did not exclude a possible variant for LpL. This could explain her initial response to O3FA, fibrate, and insulin which likely helped early in the course to induce LpL synthesis and activity. However, this regimen clearly was insufficient as the pregnancy progressed. Neither electrophoresis or genetic testing was able to provide early or definitive insight into this patient's specific defect within the lipoprotein metabolism pathway. Furthermore, the availability and access to gene therapies (of which there are currently few) is limited as studies are on-going [9]. Presently, lipidologists recommend treating the TG level rather than the genotype [22, 23]. However, differences in underlying defects among individuals may in part explain differences in response to therapy.

Several therapies have been used to manage hypertriglyceridemic pancreatitis during pregnancy (Table 1). Our patient initially received treatment with a low-fat diet, insulin, O3FAs, and encouragement for physical activity—all shown to be safe during pregnancy [24]. Medium-chain TGs, also shown to be safe in pregnancy, were introduced but not well tolerated. Glycemic control was critical through both pregnancies as insulin resistance can disrupt lipid metabolism, leading to increased production and reduced clearance of TGs, and a decrease in LpL activity [25]. Insulin was initiated for both glycemic control and stimulation of LpL activity. When her TG levels remained uncontrolled, more invasive therapies were discussed, including a heparin infusion, plasma exchange (apheresis), and total parenteral nutrition (TPN).

At the time of this patient's presentation, neither heparin nor apheresis were recommended by society guidelines. Although heparin can boost LpL activity to facilitate TG metabolism, both the Endocrine Society and American Association of Clinical Endocrinologists recommended against its use due to the risk of bleeding, hemorrhagic pancreatitis, and rebound hypertriglyceridemia [11, 26]. Recently, cases have reported successful use of infusions combining heparin with insulin for patients without bleeding tendencies [27, 28]. Apheresis was considered during the patient's hospitalization, though at that time, the American Society for Apheresis (ASFA) offered only a “weak recommendation” due to limited and low-quality evidence from observational studies and case reports [16, 29]. Since then, several case reports present apheresis as a successful tool in managing severe hypertriglyceridemia in pregnancy, particularly given its potential to remove inflammatory mediators in acute pancreatitis [30]. Apheresis can rapidly reduce lipoprotein levels to prevent acute complications, and with repeated treatments, may help prevent hypertriglyceridemic complications like pancreatitis [1, 18]. However, it requires in-hospital care, which may be impractical for many patients, and its optimal treatment frequency, along with long-term outcomes for both mother and child, remain uncertain [16, 30, 31–32]. Several case reports describe successful use of apheresis in pregnant women with elevated low-density lipoprotein (LDL) from homozygous familial hypercholesterolemia and heterozygous familial hypercholesterolemia with high atherosclerotic cardiovascular disease (ASCVD) risk, which underlies the ASFA's support for its use in these conditions. However, the ASFA continues to acknowledge the lack of evidence-based guidelines for apheresis in pregnancy, and data on its use for other types of dyslipidemia remain limited [16, 29]. As for all TG-lowering methods used in pregnancy, including apheresis, there is no data on long-term mother and child outcomes.

TPN was discussed, but deemed too invasive and impractical for outpatient care. Finally, fenofibrate and niacin were considered, though neither had been formally studied in pregnancy. Fenofibrate was selected initially as they were more frequently utilized in case reports and did not have evidence of human fetal teratogenicity at standard doses (Table 1). Furthermore, the National Lipid Association recommended their cautious use in pregnant patients with TG levels exceeding 500 mg/dL (5.6 mmol/L) [11, 33]. When the fenofibrate proved ineffective in controlling TG levels, niacin was introduced as it was discussed in available case reports and did not have any documented evidence of teratogenicity.

Niacin (vitamin B3 and nicotinic acid) is a water-soluble vitamin present in a variety of foods and supplements. It is converted by the body into its active form nicotinamide adenine dinucleotide (NAD), which plays a role in DNA and RNA synthesis and metabolism. The recommended dietary allowance is 14–16 mg/day for nonpregnant adults, and a higher dose of 18 mg/day for pregnant women in order to prevent congenital anomalies in the fetus [34, 35]. A balanced diet will easily meet this need, though prenatal vitamins commonly contain niacin. Excess niacin is stored in reserve pools or methylated by the liver to be excreted into the urine.

Niacin inhibits the hepatic enzyme hepatocyte diacylglycerol acytltransferase-2, which is critical for TG synthesis [36]. TG levels are reduced by 10%–30% in nonpregnant adults, at doses of 500–2000 mg/day. Doses up to 2000–3000 mg/day have been used [4, 37]. Niacin had been widely used to manage dyslipidemias and reduce cardiovascular events until statins became commercially available in 1987, thus, few practitioners are familiar with using niacin [38].

The main side effect of niacin is dose-dependent cutaneous flushing (reddening, burning, itching, and tingling), from niacin-mediated prostaglandin D2 release causing vasodilation. Flushing can be safely and effectively managed with the use of aspirin and ibuprofen, though the latter is not used in pregnancy [39, 40]. Rare side effects include indigestion and glucose intolerance. Hepatotoxicity has been reported with slow-release formulations [4, 41],,^42^]. Recent literature reviews recommending niacin's use are based on earlier case reports suggesting that its administration can be approached with clinical discretion, given the lack of conclusive data on teratogenicity [3, 18, 26,], as well as its necessity for normal human embryonic development [34]. The developmental delays observed in this patient's children cannot be solely attributed to niacin use during pregnancy since developmental delays are often multifactorial and difficult to explain; furthermore, both the patient's children had developmental issues, despite only one child being exposed to niacin during the patient's pregnancy [43].

Clinicians must carefully balance maternal safety with potential risks to the fetus. When this patient presented, the U.S. Food and Drug Administration (FDA) classified drugs into categories to guide their use during pregnancy (fibrates and niacin were both “Category C”); presently, drugs are now assessed by the “Pregnancy and Lactation Labeling Rule” (PLLR) which narratively outlines the risks and benefits based on available data. Both the former categorizations and present FDA guidelines state that there are no adequate studies on the use of fibrates or niacin in pregnant women [31]. Animal studies on fenofibrates have demonstrated adverse effects, including delayed delivery, decreased birth weight, increased postimplantation loss, skeletal and visceral abnormalities, abortions, and fetal deaths; [1] however, the doses administered were 7–10 times the maximum recommended human dose [44]. The available animal data on the teratogenicity of niacin is absent, with the FDA indicating that no animal reproduction studies have been conducted with niacin or with niacin extended-release tablets as of its 2023 medication labeling [45]. To date, there are no reported incidences of pregnancy associated risks or teratogenicity to niacin therapy in humans [17, 18, 31, 45].

Numerous cases of hypertriglyceridemic pancreatitis during pregnancy have been published, but none have demonstrated the effects of niacin therapy across multiple pregnancies in the same patient. In fact, the use of niacin in pregnancy remains rare due to its limited use in previously published case reports [11, 46]. A cited case demonstrating the safety of niacin describes a 39-year-old multiparous woman with a history of gestational hypertriglyceridemia and intrauterine fetal loss in her first pregnancy. During her second pregnancy, she was treated with fenofibrate 200 mg daily and niacin 500 mg four times daily starting at 12 weeks' gestation [15]. Despite this, she presented at 29 weeks with acute hypertriglyceridemic pancreatitis and TG levels of 1735 mg/dL (30.0 mmol/L), suggesting that her underlying lipid disorder differed from our patient's disorder given the ineffective response to niacin. The patient delivered a healthy female infant via cesarean section, though her pancreatitis persisted postpartum. Four days after delivery, her TG levels decreased to 591 mg/dL (6.7 mmol/L). No follow-up history was provided regarding the child's development, leaving the long-term impact of niacin on child development unclear. It is challenging to compare cases of hypertriglyceridemic pancreatitis during pregnancy given the multitude of genetic and social variables; nevertheless, clinicians should consider the use of niacin based on clinical judgement given its potential for effective TG control.

Our case highlights how initiation of niacin as early as the second trimester, with anticipatory progressive dose escalation as pregnancy progresses, can control TG levels. Based on the time course physiology of estradiol secretion and insulin resistance in pregnancy [43], we recommend monitoring serum TGs every two to 3 weeks at the start of the second trimester based on the severity of disease. Postpartum, nonhormonal, or progestin-only contraception is recommended to reduce the risk of estrogen-exacerbated hypertriglyceridemia in patients with underlying TG disorders who have experienced complications as a result of hypertriglyceridemia.

Severe hypertriglyceridemia is rare in pregnancy, but carries significant morbidity and mortality risks [2, 32, 47]. The use of niacin during pregnancy should be considered early for management as current recommendations stem from a scarcity of data rather than documented adverse events.

## Figures and Tables

**Table 1:** Commonly utilized therapeutic interventions for the management of hypertriglyceridemia in pregnancy.

Chronic outpatient management					
Lifestyle interventions	Mechanism	Effectiveness	Cost	Maternal considerations	Fetal considerations
Dietary fat restriction [18, 31] (<20% dietary fat/day)	- Reduces substrate for TG formation	- Can achieve greater than 70% TG reduction depending on dietary content [48]	- Cost effective: low cost in most scenarios	- Difficult to sustain - Paradoxical increase in VLDL and TG - Regularly monitor maternal weight	- Over-restriction may theoretically impair fetal development - Maternal malnutrition and hypoproteinemia can inhibit plasma volume expansion and reduce placental perfusion and nutrient transport - Monitor fetal growth with serial ultrasounds

Physical activity [5, 48, 49]	- Improves insulin sensitivity - Stimulates enzymatic breakdown of TGs	- Nearly 30% TG reduction depending on intensity and duration - Effective within hours	- Cost effective	- Difficult to sustain - Low to moderate activity is safe, more strenuous activity is not recommended	- Does not increase risk of miscarriage, low birthweight, or early delivery

**Medications**	**Mechanism**	**Effectiveness**	**Cost**	**Maternal considerations**	**Fetal considerations**

Niacin [17, 36, 45, 50] (18 mg–3 g/day)	- Decreases liver TG synthesis via inhibition of hepatic enzymes - Reduces fatty acid mobilization from adipose tissue - Hepatic ApoB degradation and decrease in VLDL and LDL particles	- Reduces TG by 10%–30% over weeks	- $0.20−2.00/day [51]	- May require high doses (2–3 g/day) - Minimal elevation in prothrombin time and uric acid levels - Indigestion - Hepatotoxicity with slow-release formulation - Main side effect of cutaneous flushing reduces over time and can be treated with aspirin in pregnant patients [39, 40]	- To date, no reported incidences of pregnancy associated risks or teratogenicity to niacin therapy - Inadequate human data

O3FAs [11, 14] (3–4 g/day)	- Increase LpL expression and activity in the adipose tissue - Reduces hepatic TG synthesis	- Reduces TG by 20%–60% over weeks	- Less than $1/day	- Need high doses for effectiveness - Side effects include a mild rise in LDL and fishy breath odor	- No reports of teratogenicity

Fibrate therapy [9, 11, 15, 31, 33, 52] (Gemfibrozil 600 mg BID, Fenofibrate 145–200 mg/day)	- Upregulates LpL - Decreases hepatic TG synthesis	- Reduces TG by 30%–60% over weeks to months	- Less than $1/day	- Teratogenic effects in animal studies at high doses - Inadequate human data, no current evidence of teratogenicity	- Breastfeeding contraindicated by manufacturer, but no contraindication per lipid guidelines

Medium chain TG oil [53] (Titrate as tolerated: 10mL/day, 60mL/day)	- Bypasses chylomicron formation via direct transport to liver	- Dose-dependent effectiveness	- Less than $1/day	- May provide excessive calories - Poorly tolerated due to GI symptoms: nausea, abdominal pain - Difficult to obtain, often need specialty pharmacies	- No reports of teratogenicity

Acute inpatient management					
Hospital administered therapies	Mechanism	Effectiveness	Cost	Maternal considerations	Fetal considerations
Insulin [28] (Regular insulin IV infusion at maximum tolerated rate)	- Increases LpL activity to hydrolyze TGs - Rapidly effective	- Rapidly effective in dose dependent manner but rebound can occur when therapy stopped - Up to 85% reduction in TG levels	- High cost given need for inpatient monitoring	- Potential risk of hypoglycemia, for which dextrose supplementation may be needed to maintain a blood glucose in normal 1 hr postprandial range in pregnancy: 90–140 mg (dL) (1.0–1.6 mmol/L) - Excessive dextrose is used as a substrate for TG formation - Requires frequent monitoring due to rapidly changing requirements and increased insulin resistance as pregnancy progresses	- IV infusion insulin is safe

TPN[54] (TPN pharmacist needed)	- Bypasses chylomicron formation	- Determined by fat content of TPN	- High cost given need for inpatient monitoring	- Requires expertise in TPN use - Requires IV line - Increased risk of infection and VTE	- Safe in pregnancy

Heparin [27, 28]	- Stimulates release of endothelial LpL	- 18/units per kg dose every 4–6 h, in combination with insulin, 50% reduction in TG in 24 h	- Low cost, but requires close in-hospital monitoring	- Bleeding - Lack of efficacy after repeated treatments due to depletion of LpL and rebound hypertriglyceridemia	- Maternal bleeding may impact the fetus

Apheresis [1, 16, 55]	- Plasma filtration that clears serum lipids rapidly by 37.3%–80%	- Effective, rapid lipid clearance, but does not provide sustained control and may require repeat sessions	- High cost—need for inpatient monitoring with specialty nurses and equipment - Invasive	- Not in guidelines, though encouraged in certain genetic causes of elevated LDL - Requires inpatient therapy - Iron deficiency anemia can occur	- No known fetal complications or long-term data

*Note:* Cost of medication estimated in United States Dollars ($ USD).

Abbreviations: BID, twice daily; IV, intravenous; LDL, Low density lipoprotein; LpL, lipoprotein lipase; O3FAs, omega-3 fatty acids; TG, triglycerides; TPN, total parenteral nutrition; VLDL, very low density lipoprotein.

## Data Availability

Data sharing is not applicable to this article as no datasets were generated or analyzed for this study. The information in this case report is based on individual patient data, which cannot be shared due to privacy and ethical considerations. All data have been de-identified to ensure patient privacy and comply with ethical guidelines.
